# Tooth Retention and Health Behaviors: Findings From the 4th Chinese National Oral Health Survey

**DOI:** 10.1093/geroni/igaa057.3051

**Published:** 2020-12-16

**Authors:** Xiaoyan Ou, Liwei Zeng, Yixuan Zeng, Yaolin Pei, Bei Wu

**Affiliations:** 1 Nanchang University, Nanchang, Jiangxi, China; 2 Affiliated Stomatological Hospital of Nanchang University, Nanchang, Jiangxi, China; 3 New York University, New York, New York, United States

## Abstract

This study aimed to investigate the association between tooth retention and health behaviors among Chinese older adults. Data was used from the Chinese 4th National Oral Health Survey, a national representative sample. The sample included 9054 older adults age 55-74. The control variables included age, gender, residence, region, education level, occupation, periodontal health, self-reported oral health, and self-reported general health. Logistic regression models shows that older adults who used tooth picks (OR=3.37, 95% CI: 2.94-3.85), dental floss (OR=1.93, 95%CI: 1.05-3.53), and toothpaste (OR=3.89, 95%CI: 3.14-4.83), and never smoked (OR=1.43 95%CI: 1.20-1.70) were more likely to retain more than 20 natural teeth; while having dental visit had the opposite association (OR = 0.45, 95% CI: 0.39-052). Unexpectedly, this study did not find frequency of toothbrushing was associated with tooth retention. This study suggest that improving oral hygiene and preventive dental care are key for good oral health. .

